# The Generalization Error Bound for the Multiclass Analytical Center Classifier

**DOI:** 10.1155/2013/574748

**Published:** 2013-12-26

**Authors:** Zeng Fanzi, Ma Xiaolong

**Affiliations:** Key Laboratory for Embedded and Network Computing of Hunan Province, Hunan University, Changsha 410082, China

## Abstract

This paper presents the multiclass classifier based on analytical center of feasible space (MACM). This multiclass classifier is formulated as quadratic constrained linear optimization and does not need repeatedly constructing classifiers to separate a single class from all the others. Its generalization error upper bound is proved theoretically. The experiments on benchmark datasets validate the generalization performance of MACM.

## 1. Introduction

Multiclass classification is an important and on-going research subject in machine learning. Its application is immense, such as machine vision [1, 2], text and speech categorization [3, 4], natural language processing [5], and disease diagnosis [6, 7]. Two kinds of approaches have been proposed to solve multiclass classification problem [8]. The first multiclass classification approach is extending binary classifier to handle the multiclass case directly. This included neural networks, decision trees, support vector machines, naive Bayes, and *K*-nearest neighbors. The second approach decomposes the multiclass classification problem into several binary classification tasks. Several methods are used for this decomposition: one-versus-all [9], all-versus-all [10], and error-correcting output coding [11].

The one-versus-all approach reduces the problem of classifying among *K* classes into *K* binary problems, where each problem discriminates a given class from the other *K* − 1 classes.

For the all-versus-all method, a binary classifier is built to discriminate between each pair of classes, while discarding the rest of the classes. This requires building *K*(*K* − 1)/2 binary classifiers for *K* classes problem. When testing a new example, voting is performed among the classifiers and the class with the maximum number of votes wins.

For error-correcting output coding, it works by training *N* binary classifiers to distinguish between the *K* different classes. Each class is given a codeword of length *N* according to a binary matrix *M*. Each row of *M* corresponds to a certain class.

The above multiclass classification algorithms need construct binary classifier repeatedly to separate a single class from all the others for *K* classes problem, which leads to daunting computation and low efficiency of classification. Reference [12] proposes multiclass support vector machine (MSVM), which corresponds to simple quadratic optimization and need not repeat constructing binary classifier. However, support vector machine corresponds to the center of the largest inscribed hypersphere of feasible space. When the feasible space, that is, the space of hypotheses consistent with the training data, is elongated or asymmetric, support vector machine is not effective [13]. To address the above problems, multiclass classifier based on the analytical center of feasible space (MACM) is proposed. At the same time, in order to validate its generalization performance theoretically, its generalization error upper bound is formulated and proved. And the experiments on benchmark dataset validate the generalization performance of MACM.

## 2. Multiclass Analytical Center Classifier

To facilitate the discussion of multiclass analytical center classifier, the following definitions are introduced.

**Definition 1 (chunk)**

A vector, **v** = (*v*_1_,…, *v*_*kd*_) ∈ *ℜ* ^*kd*^, is broken into *k* chunks (**v**_1_,…, **v**_*k*_) where the *i*th chunk **v**_*i*_ = (*v*_(*i*−1)∗*d*+1_,…, *v*_*i*∗*d*_).

**Definition 2 (expansion)**

Let *Vec*⁡(*x*, *i*) ∈ *ℜ* ^*kd*^ be a vector where **x** ∈ *ℜ* ^*d*^ is embedded in *kd* dimensions space by writing the coordinates of **x** in the *i*th chunk of a vector in *ℜ* ^*kd*^. 0^*ℓ*^ denotes the zero vector of length *ℓ*. Then, *Vec*⁡(**x**, *i*) can be written formally as the concatenation of three vectors, *Vec*⁡(**x**, *i*) = (0^(*i*−1)∗*d*^, **x**, 0^(*k*−*i*)∗*d*^) ∈ *ℜ* ^*kd*^. And define *Vec*⁡(**x**, *i*, *j*) = *Vec*⁡(**x**, *i*) − *Vec*⁡(**x**, *j*) as a vector where **x** is embedded in the *i*th chunk and −**x** is embedded in the *j*th chunk of a vector in *ℜ* ^*kd*^.

**Definition 3**

Given the sample (**x**, *y*) ∈ *ℜ* ^*d*^ × {1,…, *k*}, its expansion is defined as *G*(**x**, *y*) = {*Vec*⁡(**x**, *i*, *j*) | *i* = *y*, *j* = {1,…, *k*}/*i*}; the expansion of the whole sample set *S* = {(**x**_1_, *y*_1_), (**x**_2_, *y*_2_),…} is defined as *G*(*S*) = ⋃_(**x**,*y*)∈*S*_ *G*(**x**, *y*).

**Definition 4 (piecewise linear separability)**

The point sets *A* ^*i*^ ∈ *ℜ* ^*d*×*m*_*i*_^, *i* = 1,…, *k* (*k* represents the class labels and*m*_*i*_ the number of samples belonging to *i*th class), are piecewise linear separable if there exists **w** ^*i*^ ∈ *ℜ* ^*d*^, *γ* ^*i*^ ∈ *ℜ*, *i* = 1,…, *k*, where *d* represents the dimension of point, such that

(1) $$\begin{array}{c} A_{\ell }^{i}w^{i}-\gamma^{i}>A_{\ell }^{i}w^{j}-\gamma^{j},\\ i,j=1,\ldots ,k,\;\;i\neq j,\;\;\ell =1,\ldots ,m_{i}. \end{array}$$

**Definition 5 (piecewise linear classifier)**

Assume **w** = (**w**_1_,…, **w**_*k*_), where (**w**_1_,…, **w**_*k*_) ∈ *ℜ* ^*k*∗(*d*+1)^ = *ℜ* ^*d*+1^ × ⋯×*ℜ* ^*d*+1^. Given a new point **x** ∈ *ℜ* ^*d*+1^, a piecewise linear classifier is a function *f* : *ℜ* ^*d*+1^ → {1,…, *k*} as follows:

(2) $$\begin{array}{c} f\left(x\right)=\underset{i=1,\ldots ,k}{\text{argmax}}\;\;w_{i}x^{\prime }, \end{array}$$

where arg max returns to a class label corresponding to the maximum value.

To simplify the notation for the formulation of multiclass analytical center classifier, we consider an augmented weight space as follows.

Let

(3) A~ℓi=[Aℓi1]∈ℜd+1,  w~i=[wiγ]∈ℜd+1,i=1,…,k,  ℓ=1,…,mi;

then, inequality (1) can be rewritten as

(4) $$\begin{array}{c} {\tilde{A}}_{\ell }^{i}{\tilde{w}}^{i}-{\tilde{A}}_{\ell }^{i}{\tilde{w}}^{j}>0,\;i,j=1,\ldots ,k,\;\;i\neq j,\;\;\ell =1,\ldots ,m_{i}. \end{array}$$

Let $\tilde{w}=({\tilde{w}}_{1},\ldots ,{\tilde{w}}_{k})\in {ℜ}^{k*(d+1)}={ℜ}^{d+1}\times \cdots \times {ℜ}^{d+1}$. According to Definition 2, embedding ${\tilde{A}}_{\ell }^{i}$ into *ℜ* ^*k*∗(*d*+1)^ space, inequality (4) has the following form:

(5) (Vec⁡(A~ℓi,i)−Vec⁡(A~ℓi,j))w~>0,i,j=1,…,k,  i≠j,  ℓ=1,…,mi.

Consider that

(6) Vec⁡(A~ℓi,i,j)=Vec⁡(A~ℓi,i)−Vec⁡(A~ℓi,j),  i,j=1,…,k,  i≠j,  ℓ=1,…,mi.

Thus, inequality (6) can be rewritten as follows:

(7) $$\begin{array}{c} \mathrm{Vec}\left({\tilde{A}}_{\ell }^{i},i,j\right)\tilde{w}>0,\;i,j=1,\ldots ,k,\;\;i\neq j,\;\;\ell =1,\ldots ,m_{i}. \end{array}$$

Inequality (7) represents the feasible space of $\tilde{w}$ in the higher dimension space *ℜ* ^*k*∗(*d*+1)^. Similar to the binary classification based on the analytical center of version space [8], we define the slack variable $S_{i,j,\ell }=\mathrm{Vec}({\tilde{A}}_{\ell }^{i},i,j)\tilde{w}$, *i*, *j* = 1,…, *k*, *i* ≠ *j*, *ℓ* = 1,…, *m*_*i*_ and then have the following minimization problem, whose solver corresponds to the analytical center of higher dimension space:

(8) min⁡Φ(w~)=−∑ℓ=1mi ∑i≠j,i,j=1kln⁡Si,j,ℓs.t.h(w~)=12w~w~′−1=0.

In order to further simplify the formulation of multiclass analytical center classifier, we introduce some notations as follows: *M* = *k*∗(*k* − 1)∗∑_*i*=1_^*k*^ *m*_*i*_, $B=\left\{\mathrm{Vec}({\tilde{A}}_{\ell }^{i},i,j)|i,j=1,\ldots ,k,i\neq j,\;\ell =1,\ldots ,m_{i}\right\}\in {ℜ}^{M\times k*(d+1)}$; let *B*_*i*_ represent the *i*th row vector of *B*. Then, the optimization problem (8) can be rewritten as follows:

(9) min⁡Φ(w~)=−∑i=1Mln⁡Si where  Si=Biw~s.t.h(w~)=12w~w~′−1=0.

After solving the optimization problem (9) to get the optimal weight ${\tilde{w}}^{*}$, we have a piecewise linear classifier *f* : *ℜ* ^*d*+1^ → {1,…, *k*} computed in the following way:

(10) $$\begin{array}{c} f\left(x\right)=\underset{i=1,\ldots ,k}{\arg\;\max}\;\;{\tilde{w}}_{i}^{*}x^{\prime }, \end{array}$$

where argmax returns to a class label corresponding to the maximum value.

If the dataset is not piecewise linear separable, the kernel function is used to map the data into high dimension linear space.

## 3. Generalization Error Bound of Multiclass Analytical Center Classifier

In order to analyze the generalization error bound theoretically, we introduce the definition of classification margin and data radius and then deduce the margin-based generalization error bound of MACM.

**Definition 6 (classification margin)**

Given the linear classifier $h(x_{j})={\arg\;\max}_{i=1,\ldots ,k}{\tilde{w}}_{i}{\tilde{x}}_{j}^{\prime }$, the classification margin of the sample (**x**_*j*_, *y*_*j*_) ∈ *ℜ* ^*d*^ × {1,…, *k*} is defined as follows:

(11) $$\begin{array}{c} r_{j}=\underset{l=y_{j},i=\left\{1,\ldots ,k\right\}/l}{\min}\left({\tilde{w}}_{l}{\tilde{x}}_{j}^{\prime }-{\tilde{w}}_{i}{\tilde{x}}_{j}^{\prime }\right). \end{array}$$

For the whole training set *S* = {(**x**_1_, *y*_1_), (**x**_2_, *y*_2_),…, (**x**_*m*_, *y*_*m*_)}, the minimal margin is as follows:

(12) $$\begin{array}{c} \text{margi}{\text{n}}_{S}\left(h\right)=\underset{\left(x_{j},y_{j}\right)\in S}{\min}r_{j}. \end{array}$$

**Definition 7 (data radius)**

Given dataset *S* = {(**x**_1_, *y*_1_), (**x**_2_, *y*_2_),…, (**x**_*m*_, *y*_*m*_)}, the data radius is defined as follows:

(13) $$\begin{array}{c} \varsigma \left(S\right)=\underset{x_{i}\in S}{\max}||x_{i}||. \end{array}$$

**Theorem 8**

Define data radius of dataset *S* as *ς*(*S*) and data radius of dataset *Vec*⁡(*S*, *i*, *j*) as *ς*(*Vec*⁡(*S*, *i*, *j*)); if *ς*(*S*) ≤ *R*, then *ς*(*Vec*⁡(*S*, *i*, *j*)) ≤ 2*R*.

**Proof**

Consider the following:

(14) ς(Vec⁡(S,i,j)) =max⁡xl∈S⁡||Vec⁡(xl,i,j)|| =max⁡xl∈S⁡||Vec⁡(xl,i)−Vec⁡(xl,j)|| ≤max⁡xl∈S⁡(||Vec⁡(xl,i)||+||Vec⁡(xl,j)||) =max⁡xl∈S⁡||Vec⁡(xl,i)||+max⁡xl∈S⁡||Vec⁡(xl,j)||.

Because ||*Vec*⁡(**x**, *i*)|| = ||(0, **x**, 0)|| = ||**x**||, *ς*(*Vec*⁡(*S*, *i*, *j*)) ≤ 2∗max⁡_**x**_*i*_∈*S*_⁡||**x**_*i*_|| = 2*R*. This ends the proof of Theorem 8.

**Theorem 9 (see [14])**

Consider thresholding of a real-valued function *ℋ* with unit weight vectors ||**w**|| = 1 on the inner product space *X* and fix margin *r* ∈ *ℜ* ^+^. For any probability distribution *𝒟* on *X* × {−1,1} with support in a ball of radius *R* around the origin, with probability 1 − *δ* over *m* random samples *S*, any hypothesis *h* ∈ *ℋ* with *margin*_*S*_(*h*) ≥ *r* on *S* has error more than

(15) err𝒟(h)=ε(r,m,δ,R)=2m(64R2r2log⁡emr8R2log⁡32mr2+log⁡4δ),

provided *m* > 2/*ε*, 64*R* ^2^/*r* ^2^ < *m*.

From Definition 3 and inequality (7), it is shown that to correctly classify the sample *A* ^*i*^, *i* = 1,…, *k*, $G(\tilde{A})\tilde{w}=G({⋃}_{i=1}^{k}{\tilde{A}}^{i})\tilde{w}>0$ is satisfied. Here, one introduces the samples' pairs $P_{+}(A)=\{G(\tilde{A}),+1\}\in {ℜ}^{k*(d+1)}\times \{+1\}$ and $P_{-}(A)=\{-G(\tilde{A}),-1\}\in {ℜ}^{k*(d+1)}\times \{-1\}$, where 0, 1 denote the corresponding dimension vector with elements 0 or 1, respectively. So, one can construct the new samples' set *P*(*A*) = *P*_+_(*A*)⋃*P*_−_(*A*) ∈ *ℜ* ^*k*∗(*d*+1)^ × {−1, +1}.

**Theorem 10**

The binary classification of sample *P*(*A*) by analytical center classifier is equivalent to the multiclass classification of sample *A* = {*A* ^*i*^}_*i*=1_^*k*^ by multiclass analytical center classifier.

**Proof**

Assume that {*X*_*i*_^+^, *Y* ^+^} ∈ *P*_+_(*A*), {*X*_*i*_^−^, *Y* ^−^} ∈ *P*_−_(*A*), *i* = 1,…, |*P*_+_(*A*)|; then, binary classification is to solve the following feasible problem:

(16) $$\begin{array}{c} Y^{+}\left(X_{i}^{+}w+b\right)>0,\;i=1,\ldots ,\left|P_{+}\left(A\right)\right|,\\ Y^{-}\left(X_{i}^{-}w+b\right)>0,\;i=1,\ldots ,\left|P_{-}\left(A\right)\right|. \end{array}$$

Suppose the bias *b* equals 0, because *P*_−_(*A*) and *P*_+_(*A*)  are symmetrical on the origin. The feasible constraints can be rewritten as follows:

(17) $$\begin{array}{c} X_{i}^{+}w>0,\;i=1,\ldots ,\left|P_{+}\left(A\right)\right|. \end{array}$$

The feasible constraints (17) define the feasible space of weight vector **w** ∈ *ℜ* ^*k*∗(*d*+1)^; the binary classification by analytical center classifier can be formulated as follows:

(18) min⁡Φ(w)=−∑i=1|P+(A)|Xi+w>0s.t.12w′w−1=0.

Because *X*_*i*_^+^ ∈ *G*(*A*) and |*P*_+_(*A*)| = |*G*(*A*)|, the problem (18) is equivalent to problem (8). This ends the proof of Theorem 10.

**Theorem 11**

Consider the classifiers' set *ℋ* from Definition 5 with ∑_*i*=1,…,*k*_||**w**_*i*_|| = 1 on the inner product space *X*, where *h* : *ℜ* ^*d*+1^ → {1,…, *k*} and fix margin *r* ∈ *ℜ* ^+^. For any probability distribution *𝒟* on *X* × {1,…, *k*} with support in a ball of radius *R* around the origin, with probability 1 − *δ* over *m* random samples *S*, any hypothesis *h* ∈ *ℋ* with *margin*_*S*_(*h*) ≥ *r* on *S* has error more than

(19) err𝒟(h)=ε(r,m,δ,R,k)=4(k−1)m(256R2r2log⁡emr32R2log⁡32mr2+log⁡4δ)

provided *m* > 2/*ε*, 64*R* ^2^/*r* ^2^ < *m*.

**Proof**

Because the sample in *P*(*S*) is not independent, the generalization error bound cannot be attained from Theorem 9. Theorem 9 is independent of the sample distribution, so we can construct a new sample distribution *𝒟*′. According to the new distribution and dataset *S* to generate the independent sample set *P*′(*S*) with *m* samples, that is, for every (**x**, *y*) ∈ *S*, define *P*′(**x**, *y*) as the point sampled uniformly and randomly from *P*(*S*) according to the distribution *𝒟*′; then, we have *P*′(*S*) = ⋃_(**x**,*y*)∈*S*_ *P*′(**x**, *y*). From Theorem 8, the data radius *ς*(*P*′(*S*)) of *P*′(*S*) satisfies *ς*(*P*′(*S*)) ≤ 2*R*. The generalization error of hypothesis *h* over *P*′(*S*) from Theorem 9 can be calculated as follows:

(20) err𝒟′(h)=ε(r,m,δ,R)=2m(256R2r2log⁡emr32R2log⁡32mr2+log⁡4δ).

Event *E*_*i*_  which denotes a sample in *P*(*S*) is wrongly classified and event *C*  which denotes the misclassification occurs in *P*′(*S*). From the above analysis, the misclassification of any sample in *P*(*S*) causes the misclassification of the point in *P*′(*S*), so that the probability of events *E*_*i*_ and *C* satisfies the following inequality:

(21) $$\begin{array}{c} P\left(E_{i}\right)\leq P\left(C\right). \end{array}$$

Because the cardinality of *P*(*S*) equals 2(*k* − 1), the probability of sample misclassification in *P*(*S*) is written as follows:

(22) $$\begin{array}{c} P\left({⋃}_{i=1}^{2(k-1)}E_{i}\right). \end{array}$$

From union bound theorem, we have the following inequality:

(23) $$\begin{array}{c} P\left({⋃}_{i=1}^{2(k-1)}E_{i}\right)\leq \sum_{i=1}^{2(k-1)}P\left(E_{i}\right)\leq \sum_{i=1}^{2(k-1)}P\left(C\right). \end{array}$$

So the generalization error of hypothesis *h* over *S* is

(24) err𝒟(h)=ε(r,m,δ,R,k)=∑i=12(k−1)2m(256R2r2log⁡emr32R2log⁡32mr2+log⁡4δ)=4(k−1)m(256R2r2log⁡emr32R2log⁡32mr2+log⁡4δ).

This ends the proof of Theorem 11.

## 4. Computational Experiments

In this section, we present the computational results comparing multiclass analytical center classifier (MACM) and multiclass support vector machine (MSVM) [12]. A description of each of the datasets follows this paragraph. The kernel function for the piecewise nonlinear MACM and MSVM methods is *k*(**x**, **x**_*i*_) = (**x** **x**_*i*_′/*N* + 1)^*n*^, where *n* is the desired polynomial.

*Wine Recognition Data.* The wine dataset uses the chemical analysis of wine to determine the cultivar. There are 178 points with 13 features. This is a three class dataset distributed as follows: 59 points in class 1, 71 points in class 2, and 48 points in class 3.

*Glass Identification Database.* The Glass dataset is used to identify the origin of a sample of glass through chemical analysis. This dataset is comprised of six classes of 214 points with 9 features. The distribution of points by class is as follows: 70 float processed building windows, 17 float processed vehicle windows, 76 nonfloat processed building windows, 13 containers, 9 tableware, and 29 headlamps.

Table 1 contains the results for MACM and MSVM on wine and glass datasets. As anticipated, MACM produces better testing generalization than MSVM.

## 5. Summary

In this paper, the multiclass analytical center classifier based on the analytical center of feasible space, which corresponds to a simple quadratic constrained linear optimization, is proposed. At the same time, in order to validate its generalization performance theoretically, its generalization error upper bound is formulated and proved. By the experiments on wine recognition and glass identification dataset, it is shown that the multiclass analytical center classifier outperforms multiclass support vector machine in generalization error.

## Figures and Tables

**Table 1:** The generalization error of MACM and MSVM.

Dataset	Classifier	Degree of polynomial	
		1	3
Wine	M-ACM	97.74	98.65
	M-SVM	97.19	97.75

Glass	M-ACM	56.46	69.38
	M-SVM	55.14	66.15
